# Scalable and unbiased sequence-informed embedding of single-cell ATAC-seq data with CellSpace

**DOI:** 10.1038/s41592-024-02274-x

**Published:** 2024-05-09

**Authors:** Zakieh Tayyebi, Allison R. Pine, Christina S. Leslie

**Affiliations:** 1 Computational and Systems Biology Program, Memorial Sloan Kettering Cancer Center, New York, NY USA; 2 Tri-Institutional Training Program in Computational Biology and Medicine, New York, NY USA

**Keywords:** Machine learning, Computational models, Epigenomics, Software

## Abstract

Standard scATAC sequencing (scATAC-seq) analysis pipelines represent cells as sparse numeric vectors relative to an atlas of peaks or genomic tiles and consequently ignore genomic sequence information at accessible loci. Here we present CellSpace, an efficient and scalable sequence-informed embedding algorithm for scATAC-seq that learns a mapping of DNA *k*-mers and cells to the same space, to address this limitation. We show that CellSpace captures meaningful latent structure in scATAC-seq datasets, including cell subpopulations and developmental hierarchies, and can score transcription factor activities in single cells based on proximity to binding motifs embedded in the same space. Importantly, CellSpace implicitly mitigates batch effects arising from multiple samples, donors or assays, even when individual datasets are processed relative to different peak atlases. Thus, CellSpace provides a powerful tool for integrating and interpreting large-scale scATAC-seq compendia.

## Abstract (web summary)

By learning to embed DNA *k*-mers and cells into a joint space, CellSpace improves single-cell ATAC-seq analysis in multiple tasks such as latent structure discovery, transcription factor activity inference and batch effect mitigation.

## Main

Typical computational strategies to discover latent structure in scATAC-seq datasets mimic scRNA-seq workflows. First, scATAC-seq data is summarized as a sparse cell-by-event matrix, where events correspond either to an atlas of accessible peaks or to highly variable genomic tiles^1,2^, analogous to the cell-by-gene matrix in scRNA-seq analysis. The cell-by-event matrix can be binarized (1 if the event was accessible in a cell and 0 if the event was inaccessible or not captured) or contain counts. Then normalization followed by a standard dimensionality reduction method (for example, latent semantic indexing (LSI)) allows construction of a nearest neighbor (NN) graph on cells in the lower-dimensional space and use of graph-based clustering and embedding algorithms from the scRNA-seq toolkit. However, due to its high dimensionality and sparsity, dimensionality reduction and embedding of scATAC-seq is challenging and prone to complex batch effects. Another strategy summarizes single-cell chromatin accessibility profiles at the gene locus level to generate scRNA-seq-like data, allowing integration with scRNA-seq datasets^3^ but losing the representational richness of scATAC-seq.

Rather than mimicking scRNA-seq strategies, we will exploit the genomic DNA sequences underlying accessible peaks/tiles. Sequence signals, such as transcription factor (TF) binding motifs, reflect developmental state and cell identity and therefore should help reveal biologically meaningful latent structure. Importantly, we will incorporate sequence information in the latent structure discovery step of scATAC-seq analysis rather than in a post hoc analysis step. So far, few approaches have attempted sequence-informed embedding of scATAC-seq. Early work used chromVAR^4^ to represent each cell as a vector of accessibility scores relative to a fixed library of known TF motifs^5^. This approach can indeed group cells by cell type but introduces bias through a priori motif choice; moreover, TF motif accessibility scores can capture technical differences between samples and, hence, preserve batch effects. Recently, scBasset^6^ used a multitask neural network to learn both a sequence model for accessible peaks that passes through a bottleneck layer and cell-specific model vectors that predict whether a peak—given its bottleneck representation—will be accessible in the cell. This approach yields a low-dimensional representation of cells via the model vectors and assigns TF accessibility scores to cells via motif injection. However, scBasset requires training of a large neural network model where the number of tasks equals the number of cells and likely will require further optimizations to scale to large datasets. Finally, a recent method called SIMBA uses a graph-embedding approach for scRNA-seq, scATAC-seq and multiome data^7^, where cells, genes, peaks, *k*-mers and TF motifs are vertices and edges connect entities (such as peaks) that relate to other entities (such as cells). Notably, applying this method to scATAC-seq requires TF motifs to be specified before training to define the graph which could bias the learned embedding. Moreover, the cell-by-peak matrix is explicitly encoded in the graph, potentially inheriting underlying sparsity and batch effect issues.

Here, we present CellSpace, an efficient and scalable *k*-mer-based embedding algorithm for scATAC-seq. CellSpace employs a latent embedding algorithm from natural language processing called StarSpace^8^, similar to the strategy we used in the BindSpace model to learn subtle binding preferences of TFs from SELEX-seq data^9^. CellSpace learns a joint embedding of *k*-mers and cells, where cells are embedded close to each other in the latent space based on shared DNA sequence content of their accessible events. Notably, CellSpace avoids explicitly embedding peaks and tiles and, therefore, does not encode the cell-by-event matrix. Single-cell TF motif activities can be readily computed in CellSpace’s latent space; the selection of TF motifs is not required ahead of time and does not influence training. Importantly, thanks to key representational and training choices, we show that CellSpace’s sequence-aware embedding has powerful intrinsic batch mitigating properties, allowing discovery of latent structure to enable trajectory analysis and cluster discovery across multiple samples and assays, even when the individual datasets are processed independently.

## Results

### Algorithm overview

**Fig. 1:**
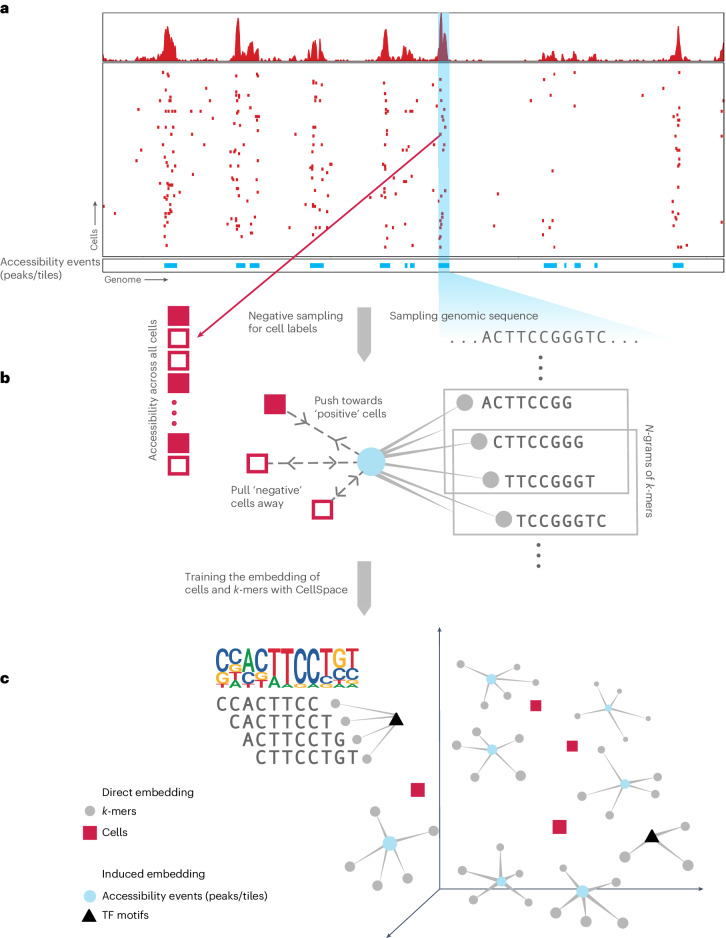
CellSpace learns a sequence-informed embedding of cells from scATAC-seq. Overview of the CellSpace algorithm. **a**, CellSpace samples sequences from accessible events (peaks or tiles) to generate training examples, each consisting of an ordered list of overlapping *k*-mers from the sampled sequence, a positive cell (where the event is open) and a sample of negative cells (where the event in closed). **b**, CellSpace learns an embedding of *k*-mers and cells into the same latent space. For each training example, the embeddings of the corresponding *k*-mers and cells are updated to pull the induced sequence embedding towards the positive cell and away from the negative cells in the latent space; learning contextual information, represented by *N*-grams of nearby *k*-mers, improves the embedding. **c**, Once the embedding of cells and *k*-mers is trained, TF motifs can be mapped to the latent space, allowing cells to be scored for TF activities based on TF-cell similarities.

CellSpace trains on scATAC-seq data to learn an embedding of DNA *k*-mers and cells into a common latent space (Fig. 1 and Methods). To generate training examples, CellSpace samples genomic sequences of fixed length from accessible events (peaks or tiles) and treats cells in which an event is present as positive labels for the sampled input sequence (Fig. 1a). This process produces left-hand side (LHS) and right-hand side (RHS) training pairs, where the LHS is a bag of *k*-mers from the sampled sequence, and the RHS is a cell in which the event is accessible. During training, CellSpace updates the embedding vectors of *k*-mers and cells to push the induced embedding representation of the LHS sequence towards the embedding of the ‘positive’ cell on the RHS and away from sampled ‘negative’ cells (Fig. 1b). Here, a *K*-negative sampling strategy^10^, where *K* negative cells are sampled at random, improves training time by updating only some of the weights at each optimization step. This technique is useful, since there are orders of magnitude that are more negative observations than positive ones, and also reduces the effect of false negatives caused by scATAC-seq sparsity. Importantly, CellSpace uses *N*-grams in the bag of *k*-mers representation to extract context from the data and improve the embedding (Fig. 1b).

Accessible events (peaks and tiles) are not explicitly embedded; an induced representation of an event can be computed from the embedding of its *k*-mers. By not directly embedding peaks and by updating the cell embedding on the basis of the *k*-mer content rather than the identity of accessible regions, CellSpace appears to be less influenced by preprocessing choices or by technical differences between batches or even assay variants. Finally, any TF motif can be embedded in the latent space based on the embedding of constituent *k*-mers from its consensus sequence (Fig. 1c). Notably, the set of (known) TF motifs to be examined is not required at training time and does not bias the embedding. Similarity between a TF motif and cell embedding in the latent space produces a TF activity score, and these motif scores are useful in characterizing cell subpopulations. Finally, similarity of cells in the latent space can be used to produce a NN graph for clustering, visualization with UMAP^11^ and other downstream analyses (Fig. 1c).

### CellSpace learns latent structure and mitigates batch effects

**Fig. 2:**
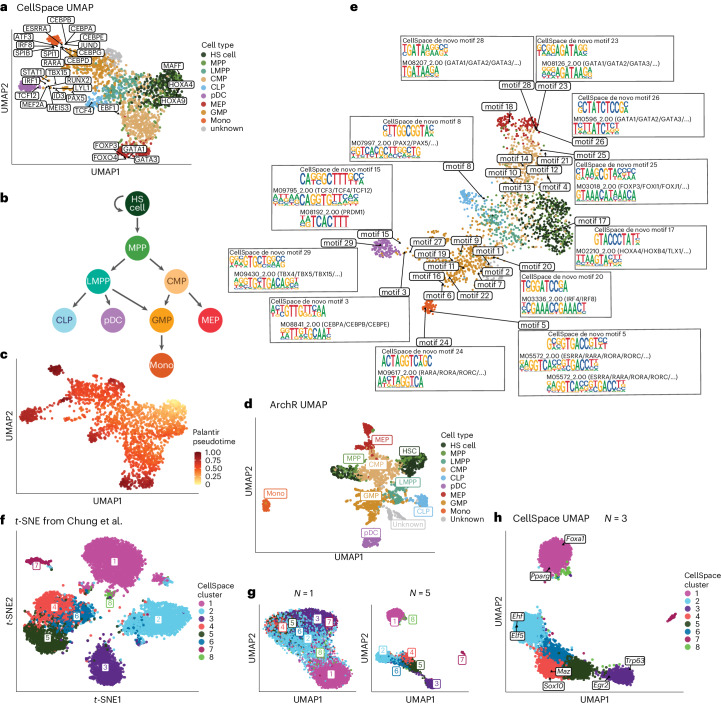
CellSpace recovers latent structure and developmental hierarchies. **a**, UMAP of CellSpace embedding for 2,154 cells from a small human hematopoietic scATAC-seq dataset annotated by fluorescence-activated cell sorting-sorted cell types. The embedding of key hematopoietic TF motifs is also shown. **b**, Current model of hematopoietic differentiation, with cell labels and colors as in **a**. **c**, Palantir pseudotime analysis using CellSpace embedding, with an HS cell starting point, identifies differentiation termini corresponding to CLP, pDC, GMP, MEP and monocyte (Mono) fates. **d**, UMAP of itLSI embedding based on cell-by-tile matrix using ArchR splits HS cell, MPP and MEP populations into two clusters due to batch effects. **e**, UMAP of cells and de novo motifs discovered based on the same trained CellSpace embedding as in **a**. DNA 10-mers that are frequent NNs of each cluster’s cells are identified and clustered by sequence content; 10-mer clusters are aligned and each converted to a PWM. **f**, Standard *t*-SNE from LSI dimensionality reduction of the cell-by-peak matrix for 7,846 cells from a murine fetal and adult mammary epithelial scATAC-seq dataset. The cells are annotated using CellSpace clusters (*N* = 3), and comparison with the original study was used to associate these clusters with cell types. **g**, UMAP of CellSpace embedding for the mouse mammary epithelial dataset shows the impact of *N*-gram parameter for *N* = 1 and 5. **h**, CellSpace with default *N* = 3 accurately captures developmental relationships between cell types. The key TF motifs in epithelial differentiation are also shown in the *N* = 3 CellSpace embedding.

**Extended Data Fig. 1:**
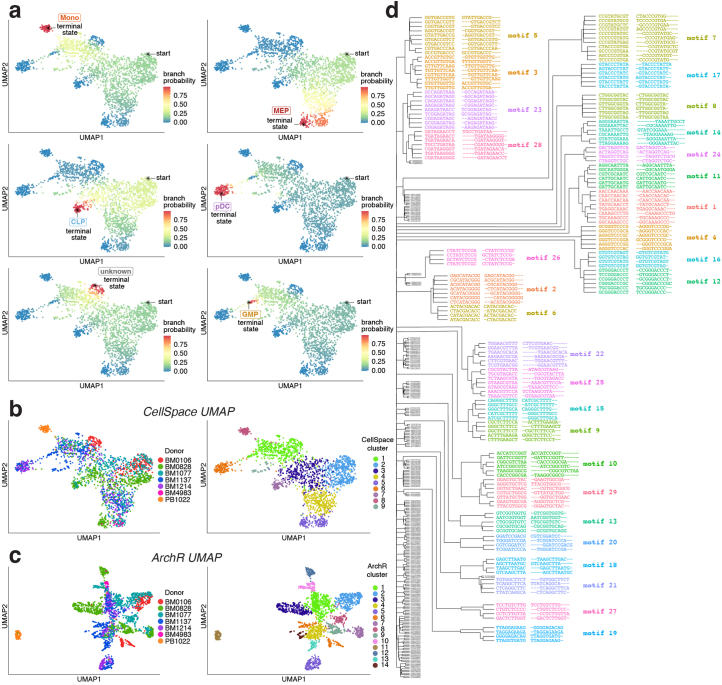
CellSpace recovers latent structure and developmental hierarchies. **a**. Palantir branch probabilities showing trajectories to 6 termini in the small human hematopoietic dataset, including termini at CLP, pDC, GMP, an ‘unknown’ GMP-adjacent population, MEP, and monocytes. **b**. CellSpace embedding annotated by donor (left) and by Seurat’s SNN-based clustering (right), which largely recovers annotated cell types. **c**. ArchR embedding of the small human hematopoietic dataset annotated by donor (left) and by Seurat’s SNN-based clustering (right). **d**. Clustering of 10-mers retrieved as frequent nearest neighbors of cell clusters from the small human hematopoietic dataset; 10-mers in each cluster are aligned and then converted to PWMs of *de novo* CellSpace motifs (visualized in Fig. 2e).

We first tested our approach on a smaller scATAC-seq dataset profiling CD34^+^ hematopoietic stem and progenitor cell (HSPC) populations from multiple human donors^5^, where ground truth cell types based on fluorescence-activated cell sorting are available. After preprocessing steps (Methods), we retained 2,154 cells for embedding with CellSpace using 50,000 variable 500-bp tiles, sampling 150-bp sequences with 3-grams of 8-mers. CellSpace obtained a biologically meaningful embedding of the hematopoietic differentiation hierarchy as visualized by UMAP (Fig. 2a), where hematopoietic stem (HS) cells and multipotent progenitors (MPPs) diverge into two main erythroid and lymphoid branches, with common myeloid progenitors (CMPs) giving rise to megakaryocyte–erythrocyte progenitors (MEPs) along one branch and lymphoid-primed MPPs (LMPPs) giving rise to common lymphoid progenitors (CLPs) along the other. The granulocyte–monocyte progenitors (GMPs) branch off both from LMPP and CMP populations, consistent with current knowledge (Fig. 2b). Trajectory analysis with Palantir^12^, using an HS cell as the origin, recovers six termini that include the most differentiated cell types represented in the dataset: CLPs, plasmacytoid dendritic cells (pDCs), MEPs, an end point within the GMP population and a GMP-adjacent population labeled as ‘unknown’ in the original study and monocytes (Fig. 2c and Extended Data Fig. 1a). We also embedded motifs for TFs important in hematopoietic differentiation using CellSpace (Fig. 2a). The location of motifs in the UMAP provides intuition for why CellSpace correctly recovers the developmental hierarchy, with cell-type-specific TFs embedded close to the cells where they are active; for example, the HOXA9 motif is embedded near the HS cell population, GATA1 near MEPs, CEBPB near GMPs, PAX5 near CLPs and IRF1 near pDCs. TFs active in multiple cell types end up in between them; for example, the ESRRA motif is close to GMP and pDC populations.

Strikingly, CellSpace mitigates batch effects in this dataset, with cells from multiple donors well mixed and with HS cell and MPP populations from three donors clustering together (Extended Data Fig. 1b). Seurat’s shared NNs (SNN)-based clustering^3,13^ on the CellSpace embedding largely recovered the known cell type labels, with earliest stem and progenitor populations HS cell and MPP grouping in one cluster (Extended Data Fig. 1b). By contrast, iterative LSI (itLSI) using ArchR separated the HS cell and MPP populations into two separate clusters based on donor and obscured the overall hierarchy (Fig. 2d and Extended Data Fig. 1c). Similarly, scBasset reported a strong donor batch effect in their embedding of this dataset, requiring a modification of the model to explicitly account for batch^6^.

We also asked whether we could learn TF motifs de novo from the CellSpace embedding, which in principle could enable the discovery of novel motifs. To do this, we used the trained CellSpace embedding to find the induced embedding of all 10-mers and compiled the 10-mers that are frequently among the NNs of cells in each cell cluster (Extended Data Fig. 1b and Methods). Next, we clustered these 10-mers on the basis of sequence composition, aligned the 10-mers in each cluster, and computed a position weight matrix (PWM) from each alignment, yielding 29 de novo motifs (Fig. 2e, Extended Data Fig. 1d and Methods). A comparison with CIS-BP^14^ motifs confirmed that the de novo motifs were similar to relevant hematopoietic TF motifs (Fig. 2e), suggesting the potential for learning novel motifs in systems where important factors are unknown.

**Extended Data Fig. 2:**
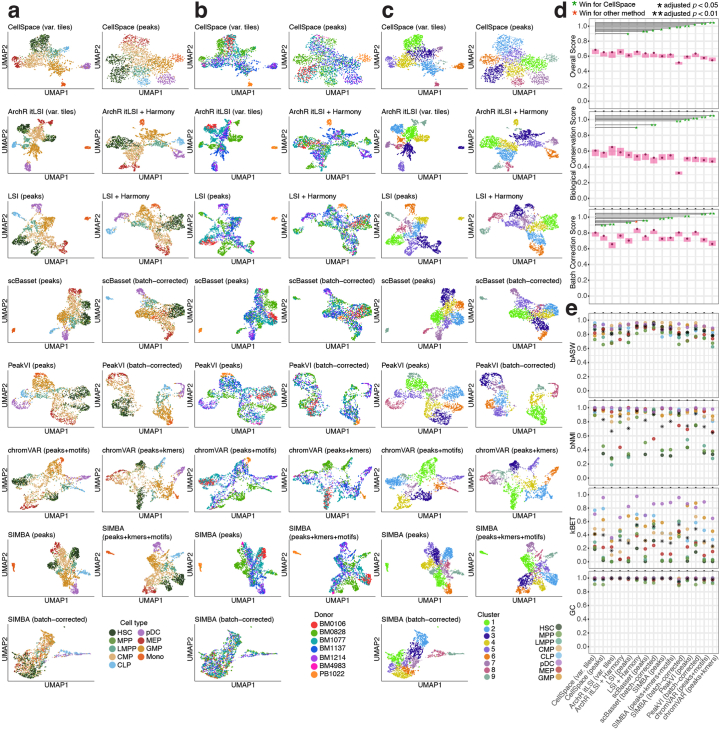
CellSpace outperforms other scATAC-seq embedding methods in batch mitigation/correction while preserving biological complexity. **a**. UMAP visualizations for multiple scATAC-seq dimensionality reduction and embedding methods on the small human hematopoietic dataset, excluding the ‘unknown’ cell type. If the method offers an explicit batch correction option, the embedding corrected for donor batch effect is labeled as ‘batch-corrected’. **b**. UMAP visualizations annotated by donor (batch). **c**. UMAP visualizations annotated by Seurat’s SNN-based cluster. **d**. Performance metrics (aggregated biological conservation score, aggregated batch correction score, and overall score) for all methods on the small human hematopoietic dataset, excluding the ‘unknown’ cell type, with 95% confidence intervals over 1000 bootstrap samples. For each metric, all methods were compared in pairwise, two-sided tests on the bootstrapping samples, under the null hypothesis that the score difference is zero. The *p*-value for each comparison was computed using confidence interval inversion, and the values were FDR-adjusted across all comparisons. Only FDR-adjusted *p*-values comparing CellSpace to other methods are shown; *: adjusted *p* < 0.05; **: adjusted *p* < 0.01. **e**. Batch correction metrics reported per cell type, excluding the monocyte cell type which consists of a single batch. Average score over all cell types is also shown.

To quantify the extent to which CellSpace implicitly corrects batch effects while preserving biological heterogeneity and to compare to other scATAC-seq embedding methods, we assessed the batch effect using published metrics (*k*-NN batch-effect test (kBET), batch average silhouette width (ASW) and graph connectivity)^15^, as well as a mutual information-based metric (batch-normalized mutual information (NMI)), and also evaluated clustering quality metrics (homogeneity, adjusted Rand index, NMI and ASW)^15,16^ (Methods). Successful batch integration should yield good batch correction metrics without sacrificing biological complexity, as assessed by the clustering metrics. To statistically assess differences in performance, we used aggregated scores—producing a single metric for batch, a single metric for biological complexity and a single overall metric—and performed a bootstrapping analysis to report 95% confidence intervals and false discovery rate (FDR)-adjusted *P* values for pairwise comparisons between algorithms (Extended Data Fig. 2a–e, Supplementary Datasets 2 and 3 and Methods). We assessed CellSpace embeddings on the basis of variable genomic tiles and on variable peaks and compared to a wide range of existing methods: ArchR’s itLSI using variable tiles; standard LSI using peaks; scBasset; SIMBA using either peaks alone or peaks, *k*-mers, and TF motifs in the graph embedding; PeakVI^17^, a variational autoencoder embedding of the cell-by-peak matrix; and chromVAR using motifs or *k*-mers. For methods that implement an explicit batch correction option (scBasset, SIMBA and PeakVI), we ran both with and without the batch covariate. For LSI-based embedding methods, we also evaluated metrics after batch correction with Harmony^18^, a widely-used single-cell integration method.

We found that CellSpace (variable tiles) significantly outperforms scBasset (with and without batch correction, adjusted *P* < 0.05 and 0.01, respectively), all variants of SIMBA (adjusted *P* < 0.05 to 0.01), PeakVI (with and without batch correction, adjusted *P* < 0.05 and 0.01, respectively), both variants of chromVAR (adjusted *P* < 0.01) and LSI (peaks) without batch correction (adjusted *P* < 0.05) (Extended Data Fig. 2d). Based on bootstrap analysis, CellSpace (variable tiles) is significantly better than ArchR itLSI (variable tiles) in terms of batch correction (adjusted *P* < 0.01), but there is no significant difference in terms of the biological complexity score and overall score between these methods. CellSpace, which uses no knowledge of batch covariates, performs comparably on this small dataset to Harmony batch correction applied to ArchR itLSI (variable tiles) or LSI (peaks). Note that the variants of LSI are not sequence-informed embeddings and do not provide batch-corrected TF motif scores.

Examining individual batch metrics by cell type (Extended Data Fig. 2e), we observed that among competing methods to CellSpace, only those with explicit batch correction improve the batch scores for HS cells and MPP, which are most affected by donor batch; in some cases (for example, ArchR itLSI + Harmony and batch-corrected SIMBA), improvement for HS cells and MPP comes at the cost of poorer performance on MEP. Overall, CellSpace (variable tiles) either ties or significantly outperforms all competing methods on this dataset, including methods with explicit batch correction, and notably outperforms sequence-informed methods that provide TF motif scores.

We found that the use of *N*-grams in CellSpace was often important for recovering well-defined latent structure in the embedding. To illustrate this effect, we applied CellSpace to a second published scATAC-seq dataset profiling 7,846 murine fetal and adult mammary epithelial cells using the published peak atlas^19^. We first reproduced the *t*-distributed stochastic neighbor embedding (*t*-SNE) visualization from the original study using standard processing of the cell-by-peak matrix to identify the reported cell types: adult luminal progenitor, adult mature luminal, adult basal, luminal progenitor-like fetal, mature luminal-like fetal and basal-like fetal (Fig. 2f). Next, we ran CellSpace with different choices of the *N*-gram hyperparameter, sampling *L* = 300 bp sequences due to the larger peak size (1,000 bp) and plotted UMAPs (Fig. 2g,h). We found that *N* = 1 (Fig. 2g, simple bag of 8-mers) yielded a diffuse embedding, while *N* = 3 (Fig. 2h, default) clarified the population structure and identified correct developmental relationships between fetal and adult cell types. The larger value *N* = 5 (Fig. 2g) began to pull cell populations further apart in the embedding, although clustering and developmental relationships were still correct. Canonical luminal (Foxa1 and Pparg) and basal (Trp63 and Egr2) TFs were correctly associated with cell populations via the CellSpace motif embeddings (*N* = 3; Fig. 2h).

### CellSpace infers single-cell TF motif activities

**Fig. 3:**
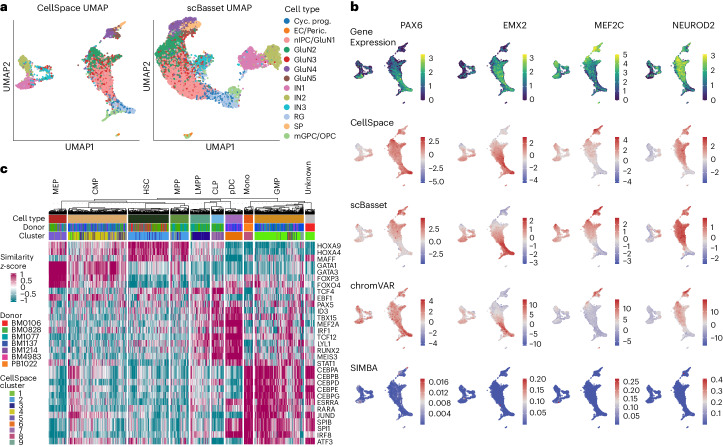
Single-cell motif scoring using CellSpace accurately maps TF activities. **a**, CellSpace and scBasset embeddings of the scATAC-seq readout of a human cortex multiome dataset with 8,981 cells. Cyc. prog, cycling progenitor; EC, endothelial cell; Peric., pericyte; nIPC, neuronal intermediate progenitor cell; SP, subplane; mGPC, multipotent glial progenitor cell. **b**, Rows show the TFs PAX6, EMX2, MEF2C and NEUROD2, overlaid on the CellSpace embedding, the gene expression for the TFs, CellSpace motif scores, scBasset motif scores, chromVAR motif deviation scores and SIMBA motif scores. **c**, CellSpace TF motif scoring for the small human hematopoietic dataset, shown as a heatmap (annotated as in Fig. 2a and Extended Data Fig. 1b).

**Extended Data Fig. 3:**
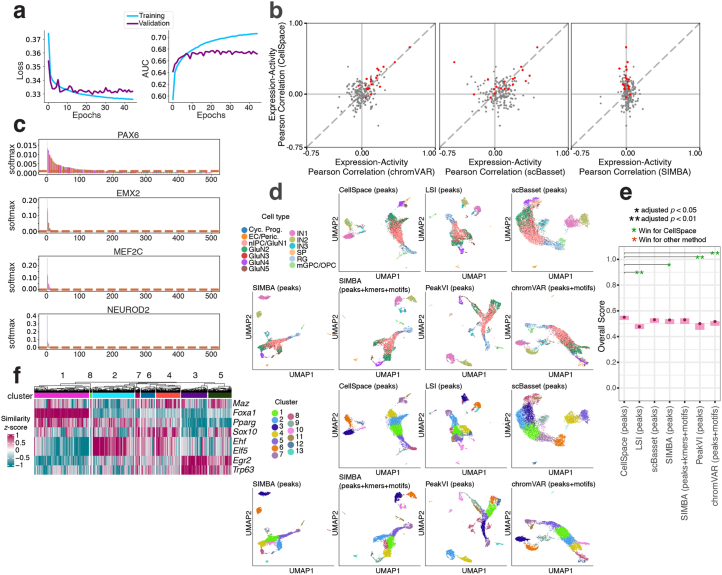
Single cell motif scoring using CellSpace accurately maps TF activities. **a**. The scBasset model training converges after 40 epochs on the human cortex multiome dataset. **b**. Comparison of CellSpace vs. scBasset TF motif activity scores, CellSpace vs. SIMBA scores, and CellSpace vs. chromVAR scores based on correlation with gene expression in the human cortex multiome dataset. Important neurodevelopmental TFs shown in red. **c**. SIMBA motif scores for PAX6, EMX2, MEF2C, and NEUROD2 can be used to rank cells and learn an association with the top-ranked cell type. **d**. UMAP embedding and Seurat’s SNN-based clustering of the human cortex multiome dataset using multiple scATAC-seq embedding methods. **e**. Overall biological conservation score for all methods on the human cortex dataset (single batch), with 95% confidence intervals over 1000 bootstrap samples. For each metric, all methods were compared in pairwise, two-sided tests on the bootstrapping samples, under the null hypothesis that the score difference is zero. The *p*-value for each comparison was computed using confidence interval inversion, and the values were FDR-adjusted across all comparisons. Only FDR-adjusted *p*-values comparing CellSpace to other methods are shown; *: adjusted *p* < 0.05; **: adjusted *p* < 0.01. **f**. TF motif scores from the CellSpace embedding for the mammary epithelial dataset (embedding and clusters visualized in Fig. 2h).

Beyond visualizing TF motifs in the CellSpace UMAP, we can compute single-cell TF activity scores via the similarity between TF motif and cell embeddings in the latent space (Methods). To systematically assess CellSpace’s motif scoring, we analyzed a recent multiome dataset profiling the human cortex containing 8,981 cells with both scRNA-seq and scATAC-seq readouts^20^. Running CellSpace with default parameters on the provided scATAC-seq cell-by-peak matrix readily captured major developmental relationships between cell types based on reported cluster annotations, with glutamatergic neuron (GluN) clusters grouping apart from inhibitory neuron (IN) clusters in the UMAP (Fig. 3a). For comparison, we ran scBasset on the same scATAC-seq dataset and found that the model converged by 45 epochs (before the default 1,000 epochs, Extended Data Fig. 3a) and trained efficiently when using specialized large-memory graphics processing units (GPUs) (Supplementary Dataset 1). Notably, scBasset applies stringent filtering to the training data, decreasing the number of peak training examples by an order of magnitude. scBasset found a topologically similar embedding to CellSpace, but unlike CellSpace and the standard LSI embedding, it failed to separate the IN cluster IN3 from the glutamatergic neurons (Fig. 3a).

Moreover, compared to TF motif scores provided by other sequence-informed embedding methods, CellSpace motif scores for key TFs correlated better with expression of the corresponding factors from the scRNA-seq readout (Fig. 3b). For example, CellSpace correctly captures that the strongest PAX6 activity is in the radial glia population, while scBasset associated PAX6 to cell populations where it is not expressed. For EMX2 and MEF2C, CellSpace better captures the overall landscape of TF activity, while scBasset overestimates activity in IN subpopulations. In other cases, such as NEUROD2, both methods correctly map the region of TF activity as validated by expression. For an overall comparison, we computed the correlation between gene expression and TF motif scores from each method for the set of important neurodevelopmental TFs identified by the original authors^20^ whose motifs passed scBasset’s filtering steps (Methods). Extended Data Fig. 3b shows that CellSpace’s motif correlation scores outperform scBasset’s scores on these neurodevelopmental factors. In particular, CellSpace TF motif scores yield positive correlation with expression for almost all these factors (17/19, upper half plane of scatterplot), in contrast with scBasset (14/19, right half plane of scatterplot), and had similar performance as chromVAR (Fig. 3b and Extended Data Fig. 3b). Finally, we trained a SIMBA embedding on the peak atlas using *k*-mers and TF motifs. SIMBA had a significantly higher memory usage than CellSpace but trained faster using peaks associated with the top principal components (Supplementary Dataset 1). The SIMBA motif scores did not provide meaningful per-cell motif activities, yielding mostly zero scores across the atlas (Fig. 3b) and near-zero correlations with TF expression (Extended Data Fig. 3b), although they could find an association with cell type via ranking (Extended Data Fig. 3c).

To compare across scATAC-seq embedding approaches, we produced UMAPs, clustered cells, computed performance scores for CellSpace and competing methods (Extended Data Fig. 3d,e, Supplementary Datasets 2 and 4 and Methods) and performed a bootstrapping analysis to report 95% confidence intervals for the overall biological complexity score and FDR-adjusted *P* values for pairwise comparisons as before. On this dataset, CellSpace (peaks) significantly outperforms LSI (adjusted *P* < 0.01), SIMBA (peaks) (adjusted *P* < 0.05), PeakVI (adjusted *P* < 0.01) and chromVAR (adjusted *P* < 0.01) but did not significantly outperform SIMBA (peaks+kmers+motifs) or scBasset (adjusted *P* = 0.087 for both) Thus, CellSpace ties or significantly outperforms all competing methods on the human cortex dataset.

Returning to the previous hematopoietic dataset (Fig. 2a), we can similarly compute motif scores for key blood developmental TFs (Fig. 3c). This analysis retrieved the correct association between TFs and HSPC populations, including GATA1 with MEP cells, ID3 with CLP and pDC cells and CEBPB with GMP cells. Interestingly, a subset of cells in the CMP population that are placed by CellSpace in cluster 1—predominantly made up of GMP cells—indeed have high CEBPB scores, suggesting progression towards the GMP cell state. Motif scoring for the mammary epithelial dataset (Fig. 2h) similarly identified correct activities of key luminal and basal TFs in fetal and adult cell populations (Extended Data Fig. 3f).

### CellSpace scales to large scATAC-seq atlases

**Fig. 4:**
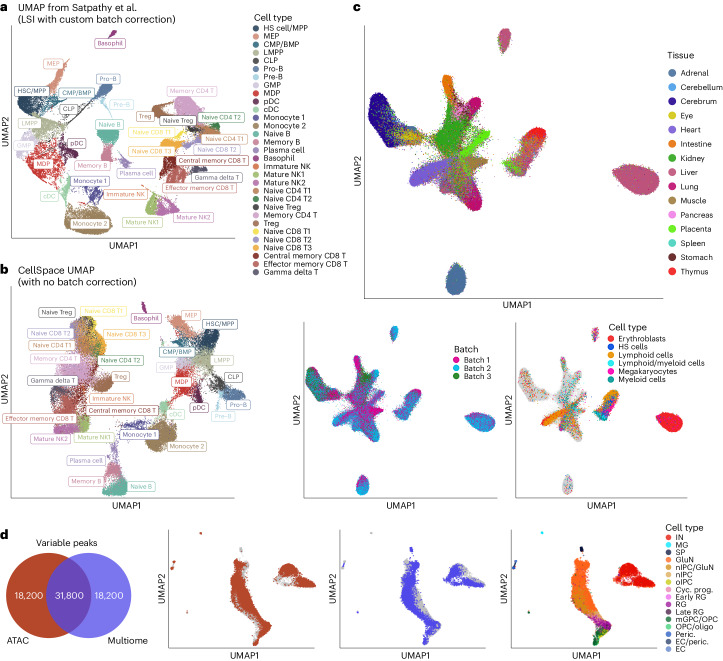
CellSpace’s embedding implicitly mitigates donor- and assay-specific batch effects in large-scale scATAC-seq datasets. **a**, UMAP of LSI dimensionality reduction with custom batch correction from original study of a large-scale multidonor human hematopoietic scATAC-seq dataset with 63,882 cells, annotated with major reported clusters. BMP, basophil–mast cell progenitor; MDP, monocyte–dendritic cell progenitor; cDC, conventional dendritic cell. **b**, CellSpace embedding of the large human hematopoietic dataset without any custom preprocessing recovers hematopoietic developmental hierarchy. **c**, UMAPs for CellSpace embedding of a human fetal tissue scATAC-seq atlas, with approximately 720,000 cells, labeled by tissue, by batch and by blood cell types across multiple tissues. **d**, CellSpace applied to human cortex chromatin accessibility data by joint embedding of two datasets: the scATAC-seq readout of the multiome dataset with 8,981 cells (Fig. 3a) and a (single-modal) scATAC-seq with 12,675 cells, processed with respect to their own peak atlases. The Venn diagram shows the top 50,000 most variable peaks from each assay, with 31,800 peaks in each atlas having nonzero overlap with the other atlas. The UMAP of the joint CellSpace embedding shows cells from each dataset, overlaid with cell type annotations from the original study. MG, microglia.

Next, to assess CellSpace’s scalability and batch-mitigating capabilities, we ran the model on several large-scale multisample datasets with challenging batch effects. First, we turned to a larger human hematopoietic dataset comprising 61,806 cells collected from bone marrow and peripheral blood from 12 healthy donors^21^, together with 2,706 cells from the smaller hematopoietic dataset^5^. The cell-by-peak matrix was originally processed in multiple steps, with LSI dimensionality reduction followed by a batch correction procedure and variable peak selection, then recomputation of LSI^21^. Cells were then clustered into 31 clusters in this final lower-dimensional space; the resulting UMAP with major clusters is reproduced here (Fig. 4a). While developmental relationships can be inferred from this embedding, there also appears to be artifactual structure from residual batch effects and noise.

**Extended Data Fig. 4:**
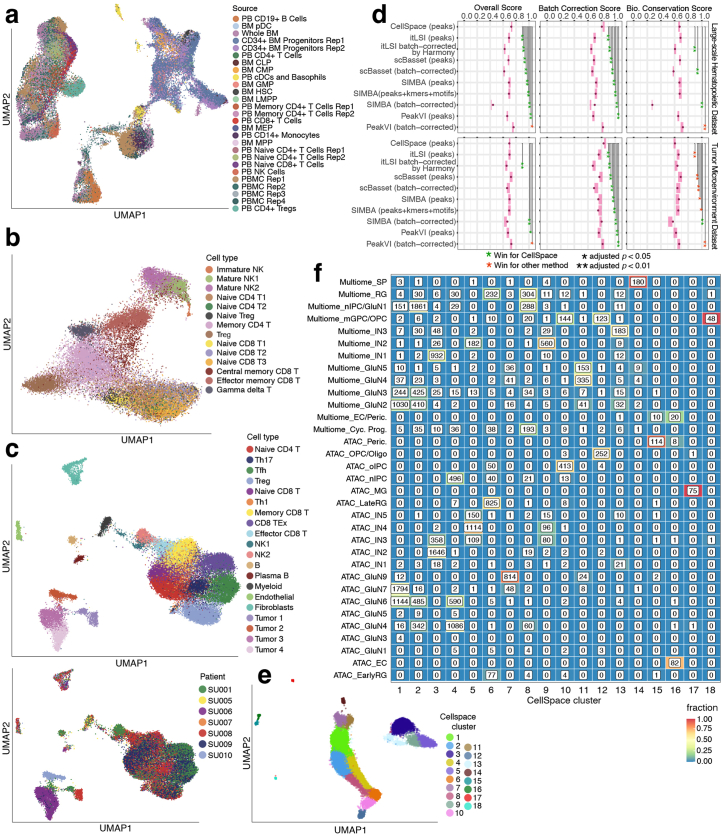
CellSpace’s embedding implicitly mitigates donor- and assay-specific batch effects in large-scale scATAC-seq datasets. **a**. Batches and human donors are well mixed in the CellSpace embedding of the large human hematopoietic dataset (visualized in Fig. 4b). **b**. CellSpace embedding of the large human hematopoietic dataset restricted to 30,211 natural killer and T cells. **c**. CellSpace embedding of 37,818 cells from a basal cell carcinoma TME scATAC-seq dataset from 7 patients, annotated by cell type and by donor, recovers immune and stromal cell types with no evident donor batch effect. **d**. Performance metrics (aggregated biological conservation score, aggregated batch correction score, and overall score) for all methods on the large human hematopoietic and TME datasets, excluding the tumor clusters, with 95% confidence intervals over 1000 bootstrap samples. For each metric, all methods were compared in pairwise, two-sided tests on the bootstrapping samples, under the null hypothesis that the score difference is zero. The *p*-value for each comparison was computed using confidence interval inversion, and the values were FDR-adjusted across all comparisons. Only FDR-adjusted *p*-values comparing CellSpace to other methods are shown; *: adjusted *p* < 0.05; **: adjusted *p* < 0.01. **e**. Seurat’s SNN-based clustering after CellSpace joint embedding of the (single-modal) scATAC-seq and the scATAC-seq readout of the multiome human cortex datasets. **f**. Membership of annotated cell types from multiome and (single-modal) scATAC-seq human cortex datasets in CellSpace clusters as shown in **e**, after joint embedding, showing coherent clusters with membership from both assays.

We asked whether CellSpace’s *k*-mer-based embedding could overcome batch effects and find latent structure without multiple custom preprocessing steps. We therefore ran CellSpace on this approximately 63,000 cell dataset using the cell-by-peak matrix for the top 50,000 variable peaks and with default parameters, except for increasing the embedding dimension and number of epochs (Methods). Here, we exploit the fact that CellSpace is memory-efficient even for large-scale datasets (Supplementary Dataset 1), since random training examples are generated at every step of optimization and only the sparse count matrix and its corresponding genomic sequences are indexed and stored in memory (Methods). A UMAP visualization shows that CellSpace faithfully captured the hematopoietic developmental hierarchy within the HSPC compartment and correctly linked progenitor populations to more mature blood cell types (Fig. 4b); for example, the monocyte–dendritic progenitor population was embedded near to monocytes and conventional dendritic cells, while CLP cells displayed a differentiation trajectory towards pro-B and pre-B cells. We also found that batches and donors were well mixed in the embedding (Extended Data Fig. 4a). Given the diversity of this dataset, we were able to obtain more resolution by retraining the CellSpace embedding on specific compartments, for example, to reveal detailed relationships among natural killer and T cell populations (Extended Data Fig. 4b).

We further applied CellSpace to a scATAC-seq dataset profiling the tumor immune microenvironment (TME) in basal cell carcinoma biopsies from seven patients^21^, comprising 37,818 cells. Although the authors reported a detectable batch effect that confounded further analyses and required attenuation^21^, we ran CellSpace directly on 50,000 variable peaks and recovered the identified T cell types as well as other lymphoid, myeloid, endothelial and fibroblast populations that were well mixed over donors (Extended Data Fig. 4c). As has been described in tumor scRNA-seq analyses, the cancer cells from different patients retained more distinct identities in the embedding.

We again assessed CellSpace’s batch mitigation properties by comparing biological complexity, batch correction and overall metrics against both sequence-informed and sequence-ignorant methods, with and without explicit batch correction, through bootstrap analysis (Extended Data Fig. 4d, Supplementary Datasets 2 and 5 and Methods). An important caveat here is that the reported labels themselves are somewhat uncertain, since the authors had to perform a difficult batch correction and clustering to annotate their dataset. Nevertheless, for the large hematopoietic dataset, CellSpace significantly outperformed (adjusted *P* < 0.01) all methods except for PeakVI (batch corrected), which outperformed CellSpace here (adjusted *P* < 0.05), even though it was one of the poorer performers on the hematopoietic and cortex datasets. The performance improvement was due to PeakVI’s better biological complexity score relative to reported cell type labels (adjusted *P* < 0.01); the batch correction scores for CellSpace were higher than PeakVI but not significantly different.

For the TME dataset, CellSpace significantly outperformed all other methods based on batch score (adjusted *P* < 0.01 in all cases) but only outperformed batch-corrected SIMBA on biological complexity score (adjusted *P* < 0.01), with comparison to other methods giving ties or losses for this score. On overall score, CellSpace mainly gave statistical ties to other methods, with significant wins over Harmony-corrected itLSI (adjusted *P* < 0.01), batch-corrected SIMBA (adjusted *P* < 0.01) and PeakVI (adjusted *P* < 0.05) but a loss to batch-corrected PeakVI (adjusted *P* < 0.05) (Extended Data Fig. 4d and Supplementary Datasets 2 and 6). We note, however, that PeakVI does not provide TF motif scores, and no other sequence-informed method (that is, with the potential to compute batch-corrected single-cell motif scores) outperforms CellSpace.

To demonstrate scalability up to another order of magnitude in number of cells, we applied CellSpace to a very large, diverse and multidonor human fetal scATAC-seq atlas^22^, consisting of approximately 720,000 cells from 20 donors in three batches. We used a latent space of dimension 70 to accommodate the diversity of cell types, computed variable peaks on a sample of approximately 5% of cells and used these events to train the full-scale embedding without difficulty (Methods and Supplementary Dataset 1). Qualitative visualization with UMAP showed proximity between more closely related tissues (Fig. 4c), and batches were well mixed. Moreover, blood cell types from multiple organs clustered together, with lymphocytes from thymus and cells labeled ‘lymphoid/myeloid’ from the placenta in the same cluster (Fig. 4c).

Finally, we applied CellSpace to combine two distinct datasets using different assays to profile the human cortex: the scATAC-seq readout of the multiome dataset presented above (Fig. 3a) and a single-modal scATAC-seq dataset from the same study^20^. These two datasets were processed independently to generate different peak atlases. Selecting the 50,000 most variable peaks in each dataset yielded only 31,800 peaks (‘shared’ peaks) with nonzero overlap but not necessarily the same boundaries (Fig. 4d). Without reprocessing these datasets to generate a combined cell-by-peak matrix relative to a common peak atlas, this situation would yield an ‘uncorrectable’ batch effect for standard methods. We trained a CellSpace embedding to successfully integrate the two datasets, each represented with respect to its own peak atlas and associated with a batch covariate, which we used to avoid pushing cells from different batches away from each other in negative sampling (Methods and Fig. 4d). The combined embedding recovered the correct overall structure based on cell type annotations from each dataset (Fig. 4d), with inhibitory and glutamatergic neurons well separated and progenitor populations, such as oligodendrocyte progenitor cells (OPCs) and radial glia (RG), placed at the apex of the developmental manifold. Clustering on the CellSpace embedding identified coherent clusters that mixed cells of similar types from the two datasets (Extended Data Fig. 4e,f). This example shows the unique and powerful ability of CellSpace to integrate independently processed chromatin accessibility datasets through its sequence-informed embedding.

## Discussion

By training an embedding of both DNA *k*-mers and cells into a common latent space with a memory-efficient implementation, we have shown that CellSpace learns latent structure in multisample and even multiassay scATAC-seq datasets while mitigating batch effects. The TF motif activities in single cells can naturally be inferred on the basis of the similarity of TF motif and cell embeddings in the latent space, without requiring the TF motifs to be known at training time. In the large multibatch datasets shown here, CellSpace’s sequence-informed embedding implicitly mitigated batch effects, even without use of a batch covariate. In one case, where datasets were independently processed with respect to distinct peak atlases, we used a batch covariate simply to avoid pushing cells from separate batches away from each other in training; this strategy allowed us to correct a batch effect that would be ‘uncorrectable’ by other methods without reprocessing from scratch. Indeed, we have found only rare cases where a clear batch effect persists after training CellSpace. In such cases, Seurat’s anchor-based data integration method^3^, inspired by mutual NNs^23^, can be readily applied to the CellSpace embedding for batch correction (Methods).

CellSpace was overall a top performer in benchmarking across datasets, giving equal or significantly better performance compared to standard LSI-based methods with or without Harmony batch correction or to other sequence-based embedding methods. Importantly, no other sequence-informed method—that is, with the potential to compute batch-corrected single-cell motif scores—outperforms CellSpace. CellSpace has impressive batch mitigation properties, with only one loss to another method in all pairwise comparisons across three datasets, while achieving a favorable tradeoff with biological complexity metrics. While explicit batch correction (for example, by Harmony) sometimes helps and sometimes hurts (it is not always clear which is happening), CellSpace gives consistently strong performance without the requiring an explicit consideration of batch effects.

We have found that the default parameters (Methods) work well in most cases, but hyperparameter tuning is sometimes needed; for example, a very large and diverse dataset typically requires a higher dimensional embedding space and a larger number of epochs to train. A qualitative sign that CellSpace hyperparameters need to be optimized—or possibly that longer training is needed—is a ‘cloudy’ UMAP visualization, where distinct cell types or states have not been pulled apart enough. We have found it easier to obtain a good embedding with minimal changes to default parameters when using variable tiles rather than a peak atlas; the peak atlas quality may influence the amount of parameter optimization required. Using top variable peaks or genomic tiles identified by itLSI markedly improves running time while preserving or possibly improving the embedding quality. We found that Seurat’s SNN-based clustering on the CellSpace embedding often required a higher resolution to obtain the same number of clusters as compared to a standard itLSI-based embedding. Additionally, the batch-aware version of CellSpace, where negative cells are sampled within the same batch as the positive cell, appears to be broadly useful for integrating datasets, whether processed with respect to different peak atlases or when using variable tiles.

We foresee an extension of CellSpace to multiome data where cells, genes and *k*-mers are embedded in the same space, and cell embeddings are updated both by sampling sequences from peaks and by expression-weighted gene lists. This will entail weighting how much sequence versus gene expression features should influence the cell embedding. We note that StarSpace has also been reformulated as a graph-embedding problem, where entities are vertices and (LHS, RHS) pairs specify edges in a graph^24^, and used by SIMBA for embedding scRNA-seq, scATAC-seq and multiome data^7^. For scATAC-seq, cells, peaks, *k*-mers and TF motifs are all explicitly embedded as vertices, and each cell is connected by edges to its peaks. While related to our approach, CellSpace makes important algorithmic choices that are less naturally framed as a graph-embedding problem. In particular, CellSpace does not explicitly embed peaks (which appears to mitigate batch effects in datasets analyzed here), uses negative sampling to address the label asymmetry in scATAC-seq, employs *N*-grams to capture local sequence context and uses sampling of sequences from accessible events to improve robustness. Finally, CellSpace enables the embedding of DNA sequences that were not explicitly introduced during training and importantly does not rely on any a priori choice of motifs.

There is also a connection between CellSpace and scBasset. We can view CellSpace as implicitly embedding peak (sub)sequences to a latent space while representing every cell as a classification model that predicts whether the embedded sequences are accessible in that cell, based on the cosine similarity between the sequence and cell in the latent space. This view is made explicit in scBasset, which learns a neural network embedding of peak sequences together with cell-specific model vectors in the latent space and minimizes classification loss using the entire cell-by-peak matrix as output labels. The neural network sequence embedding is not only more expressive than our *N*-gram of *k*-mers representation but also may be more prone to overfitting and learning batch-specific technical artifacts (which are explicitly modeled). Additionally, scBasset requires high-memory GPUs to train the neural network model in a practical running time. Finally, scBasset’s multitask classification approach may be susceptible to asymmetric label noise in the binary cell-by-peak matrix, that is, false negatives not captured in the library. Still, these sequence-informed embedding methods—CellSpace, graph embedding and neural network—potentially have complementary strengths that could be combined in future algorithmic innovations for discovery of latent structure in single-cell epigenomic data.

We note several current limitations of CellSpace. As described above, CellSpace is for now restricted to embedding scATAC-seq data and does not handle other single-cell assays or co-assays such as multiome, although such extensions are possible. Our current consensus *k*-mer approach to motif embedding, which enables motif activity scoring via similarity with cell embeddings in the latent space, is fairly simple and may not be suitable for composite motifs. More sophisticated approaches could be explored, such as representing the motif using *N*-grams of *k*-mers or as a weighted ensemble of matching sequences rather than a single consensus sequence. Finally, some amount of parameter tuning, for example, the dimension of the latent space and the number of training epochs, may be required to obtain a useful embedding. Beyond the heuristics for parameter choice provided here, we hope in the future to develop intrinsic metrics of embedding quality to enable automation of the parameter search.

## Methods

### CellSpace algorithm

CellSpace uses the StarSpace (mode 0) algorithm^25^ to learn a co-embedding of DNA *k*-mers (*k* = 8 by default) and cells into a latent vector space ${{\mathbb{R}}}^{d}$ (*d* = 30 dimensions by default) based on training example sequences sampled from accessible events.

Accessible events are either an atlas of accessible peaks or variable tiles, for which a cell-by-event matrix of accessibility is available. Top variable tiles (500 bp genomic bins) can be identified using ArchR’s itLSI method. When stated, we used top variable peaks instead of the entire peak atlas, which were identified with an adaptation of ArchR functions.

Starting from a binary cell-by-event matrix, CellSpace creates multiple training examples per event (20 by default) while training during each epoch (50 epochs by default). To generate a training example for an event, an *L*-length (*L* = 150 bp by default) DNA sequence is randomly sampled from the corresponding genomic region. The bag of *L* − *k* + 1 consecutive overlapping *k*-mers, created by sliding a window of size *k* across the sampled sequence by one nucleotide at a time, is used as the ‘input’. Assuming each DNA *k*-mer and its reverse complement have identical genetic information, we hash them to the same row of the embedding matrix. The cells for which the event is accessible are used as ‘positive labels’. The model is optimized so that the ‘input’ sequence is embedded closer to its ‘positive labels’ in the latent space than to ‘negative labels’ (that is, *K* randomly sampled cells for which the event is not accessible) which are selected by *K*-negative sampling.

StarSpace represents features, which are embedded directly, and entities (that is, bag of one or more features) by a *d*-dimensional vector. The inferred embedding of an entity composed of *M* features is given by $\frac{1}{{M}^{P}}\mathop{\sum }\nolimits_{i=1}^{M}{\mathbf{w}}_{i}$, where **w**_1_,⋯,**w**_*M*_ are the vector representations of its features and *P* = 0.5 is the default value. CellSpace embeds cells (as ‘labels’) and *k*-mers (as features in ‘input’) directly and infers the embedding of any DNA sequence as a bag of *k*-mers, enabling the comparison of sequences and cells in the same space.

Additionally, CellSpace learns contextual information from the relative position of the *k*-mers by training StarSpace with *N*-grams (window of *N* = 3 consecutive *k*-mers by default), so that each pair of *k*-mers within an *N*-gram is also considered as a feature, embedded directly with a row in the embedding matrix and added to the ‘input’ of the training example. For *N* > 1, StarSpace uses a hashing trick to retrieve the embedding vector of an *N*-gram. The user can control the size of the hashing map ‘bucket’.

At step *i* of stochastic gradient descent optimization, StarSpace picks one random ‘positive label’ as the right-hand side entity RHS_*i*_ of the training example and uses the ‘input’ as the left-hand side entity LHS_*i*_. CellSpace randomly selects a positive cell for the corresponding event as the RHS_*i*_. The ‘input’ *L*-length sampled sequence represents the LHS_*i*_, and its embedding is inferred from the embedding vectors of its features as described above. CellSpace then samples *K* random ‘negative’ cells ${\mathrm{c}}_{{n}_{1}}$ … ${\mathrm{c}}_{{n}_{K}}$—for which the event is not accessible—and optimizes the parameters to pull the LHS_*i*_ closer to the embedding of the positive cell and away from that of the negative cells by minimizing the margin ranking loss, as shown in

$${{\mathrm{Loss}}}_{i}=\frac{1}{K}\mathop{\sum }\limits_{k=1}^{K}\max \left(0,{\rm{margin}}-{\mathrm{sim}}({{\mathrm{LHS}}}_{i},{{\mathrm{RHS}}}_{i})+{\mathrm{sim}}({{\mathrm{LHS}}}_{i},{\mathrm{c}}_{{n}_{k}})\right).$$

Here, ‘sim’ is the cosine similarity in the embedding space by default. Therefore, the loss increases unless the event is closer to the positive cell than the negative cell, and the difference is greater than the margin. The embedding of a negative cell is not updated if it yields zero loss, because it is already sufficiently distant to the event.

CellSpace has been integrated into the C++ StarSpace implementation so that the sparse cell-by-event matrix and the DNA sequences of the events are loaded, parsed, indexed and stored in memory. Training example batches are randomly created in real time during training and are only temporarily stored, so that the running time of CellSpace will increase linearly with the number of training examples and the memory usage is constant. Furthermore, CellSpace utilizes the parallel training capability of StarSpace, which enables scalability to larger single-cell ATAC-seq datasets.

Multiple scATAC-seq datasets represented by different sets of events (that is, peak and tile sets) can be simultaneously embedded by CellSpace. All datasets are initially loaded, and training examples are created in random order. The event, the positive cell and the negative cells for each training example are sampled from the same dataset. This co-embedding utilizes the shared DNA sequence information between events that may not have the exact same genomic region.

### CellSpace visualization, clustering and motif embeddings

CellSpace outputs embedding vectors for cells and *k*-mers after training a StarSpace model on scATAC-seq data.

The CellSpace embedding of each TF motif is computed by creating a bag of *k*-mers by sliding a *k* bp window across the consensus motif sequence, then computing its embedding from the embedding vectors of its length(motif) − *k* + 1 constituent *k*-mers as previously described for a StarSpace entity. Cell-by-TF similarities (that is, cosine similarity between CellSpace embedding vectors) are computed and *z*-scored across all cells per TF to represent TF activities.

The pairwise distance matrix of cells (that is, cosine distance between CellSpace embedding vectors) is used to build a NN and SNN graph. Cells are visualized with a UMAP embedding and clustered using the Louvain method on the SNN graph by Seurat (v.3 or higher)^3,26^.

To visualize cells and TFs in the same space, the embedding vectors of selected TFs are concatenated to the embedding vectors of cells, and their pairwise cosine distances are used to compute a UMAP embedding as described above.

The sequence-informed embedding of CellSpace captures the structure of scATAC-seq data across multiple samples, donors and datasets while mitigating possible batch effects. However, if a batch effect persists in the CellSpace embedding, we found the problem could be easily corrected by Seurat’s anchor-based data integration method^3^. CellSpace can place multiple datasets in a shared low-dimensional space, which can be used instead of canonical correlation analysis to identify and score pairs of mutual NNs ‘anchors’ between datasets. Similarly, the NN graphs used for weighting the anchors for cells within each dataset can be created from the CellSpace embedding, instead of using principal component analysis dimensionality reduction. Finally, the batch effect can be removed by correcting the CellSpace embedding of ‘query’ datasets with respect to the ‘reference’ dataset, similar to how gene expression matrices are corrected by Seurat.

### Discovering de novo motifs with CellSpace

We computed the inferred embedding of all possible DNA 10-mers by sliding an 8 bp window across each 10 bp sequence and computing the average CellSpace embedding of its three constituent 8-mers. We built a bipartite *K* = 50 NN graph between cells and 10-mers on the basis of their cosine distance in the embedding space, representing each 10-mer and its reverse complement as a single vertex in the graph.

For each group of cells, we identified the 10-mers that were among the NNs of at least 20% of its cells. These 10-mers were clustered by kmer::cluster (v.1.1.2) in R^27^, using a top-down tree-building approach and cutting the tree at height of 0.5. For each cluster of size greater than three, we aligned the 10-mers by msa::msaClustalW (v.1.26.0) in R with default settings^28^. From each alignment, we computed the PWM of a de novo motif. The embedding of each de novo motif was computed as the average embedding of the 10-mers in its corresponding cluster.

### Evaluating scATAC-seq analysis results

#### Clustering and visualization

For each embedding, the cells were clustered using Seurat^26^ v.4.3.0 (SNN-based method) and visualized by UMAP, with *K* = 20 by default and the metric set to ‘cosine’ for CellSpace and to ‘euclidean’ for other methods. We used a range of values as Louvain clustering resolution and picked the value that yielded the same number of clusters as cell types (that is, the cell labels that would be used as ground truth in evaluation). In a few cases where no such value was found and there were too many clusters, we merged the smallest clusters into the nearest larger clusters based on their connectivity in the SNN, using the R function CellSpace::merge_small_clusters which was adapted from Seurat::GroupSingletons.

#### Biological conservation scores

To evaluate the embedding and clustering results from each method, we used the implementation of ASW, NMI and the adjusted Rand index by scib^15^ v.1.1.3 in Python, as well as the implementation of homogeneity by scikit-learn^16^ v.1.3.0 in Python. The biological conservation score was computed as the average of all four metrics.

#### Batch correction scores

To evaluate the batch effect in the embedding of each method, we used batch ASW, graph connectivity and kBET from scib. To speed-up the bootstrapping process for the large-scale hematopoietic and tumor microenvironment datasets, we used the implementation of kBET by scib-metrics v.0.3.3 in Python, which approximates the method used in the original scib package and utilizes GPUs. The metric batch NMI was computed as 1 − NMI (cluster and batch) in each cell type and reported as the average over all cell types. The batch correction score was computed as the average of all four metrics.

#### Overall score

The overall score is the weighted average of the biological conservation and batch correction scores, with 0.6 and 0.4 as their relative weights, respectively.

#### Bootstrapping

For each dataset, we created *B* = 1,000 bootstrap samples from the original dataset by resampling the same number of cells, with replacement. For each embedding, we clustered every bootstrap sample and computed the corresponding benchmarking scores as described above. For confidence level 1 − *α* of a statistic, we reported the percentile confidence interval, that is, the $\frac{\alpha }{2}$ and $1-\frac{\alpha }{2}$ quantiles of the bootstrap distribution. To compare the scores of two methods, we performed a two-sided test under the null hypothesis *θ* = 0, where *θ* is the difference in scores. We computed the *P* value of the null hypothesis using a confidence interval inversion; the *P* value for a two-sided test of the point-null hypothesis *θ* = *θ*_0_ is the smallest $\alpha \in [\frac{1}{B},1]$, such that ${\theta }_{0}$ is not contained in the 1 − *α* confidence interval from the bootstrap distribution of *θ*. For each dataset, we performed pairwise tests between all the methods and FDR-adjusted the *P* values.

#### Dataset-specific benchmarking details

For the small hematopoietic dataset, the ‘unknown’ cell type was included in the embedding but excluded from benchmarking evaluations. For the TME dataset, to reduce potential label uncertainty, we restricted the evaluation of clustering and batch correction metrics to the nontumor cells, although all cells were embedded by all methods.

Dataset-specific and method-specific embedding and benchmarking details and hyperparameters are provided in the Supplementary Note.

### Cellspace and other method parameters

#### ArchR

We used ArchR^2^ v.1.0.1 and its implementation of itLSI to identify the most variable tiles (genome-wide 500-bp bins) and used the dimensionality reduction from the last iteration of itLSI as the ArchR embedding. For batch correction, we used Harmony^18^ v.0.1.1.

#### scBasset

scBasset^6^ v.0.1 was trained with its default Basenji-inspired architecture and a bottleneck layer size of 32. For batch correction, batch labels were provided as input to the scBasset-BC architecture, which adds a fully connected layer to predict the batch-specific contribution before the final sigmoid.

#### SIMBA

For the peak-only version, SIMBA^7^ v.1.2 was run on peak-by-cell matrices using default settings. Unless stated otherwise, the embedding was trained on peaks associated with top PCs. For the sequence-aware version, the peak set was annotated with *k*-mers and motifs using the scan_for_kmers_motifs R function, and peak-motif and peak-kmer edges were included in graph generation. To obtain motif scores, we used the compare entities function between cell embedding and motif embedding matrices, followed by subsequent softmax transformation. For batch-corrected SIMBA, peak-by-cell matrices were split by batch. The edges between batches were inferred using their mutual NN implementation in the infer_edges function, and the edges between batches were included in graph generation. For all versions, the model was trained for the recommended ten epochs, at which point the validation loss leveled and the embedding had converged.

#### PeakVI

PeakVI^17^ (scVI-tools v.1.0.0) was run with default settings (two encoder layers, two decoder layers and a dropout rate of 0.1) on the peak-by-cell matrix as input and optionally providing donor annotations for explicit batch correction.

#### chromVAR

We used chromVAR^4^ v.1.16.0 to compute ‘deviations’ of JASPAR 2020 motifs^29^ for the motif version, or that of DNA 8-mers for the *k*-mer version, from the peak-by-cell count matrix, following standard steps with default parameters. Highly correlated features (cor > 0.9) and features with low variance (s.d. < 1.5) were removed from the cell-by-motif/kmer deviation *z*-score matrix, and a principal component analysis was performed on the filtered matrix. The PCs were used as the chromVAR embedding.

#### CellSpace

By default, CellSpace samples *L* = 150 bp sequences, uses 8-mers with 3-grams (*k* = 8 bp, *N* = 3), generates 20 training examples per event (tile or peak) per epoch and trains for 50 epochs to learn a *d* = 30-dimensional latent space representation of cells and *k*-mers. To extract peak and tile sequences from reference genomes, we used GenomicRanges v.1.46.1, Biostrings v.2.62.0 and BSgenome v.1.62.0 in R.

The dataset-specific preprocessing steps and hyperparameters for CellSpace and other methods are detailed in Supplementary Note.

### Reporting summary

Further information on research design is available in the Nature Portfolio Reporting Summary linked to this article.

## Online content

Any methods, additional references, Nature Portfolio reporting summaries, source data, extended data, supplementary information, acknowledgements, peer review information; details of author contributions and competing interests; and statements of data and code availability are available at 10.1038/s41592-024-02274-x.

### Supplementary Information

Supplementary information Details about data preprocessing and running each model on different datasets.

Reporting Summary

Supplementary Dataset 1 Run time and memory usage information for all datasets.

Supplementary Dataset 2 Biological conservation, batch correction and overall performance scores.

Supplementary Dataset 3 Result of pairwise comparisons of performance metrics between all methods over 1,000 bootstrap samples, including FDR-adjusted *P* values, for the small human hematopoietic dataset (Buenrostro et al.).

Supplementary Dataset 4 Result of pairwise comparisons of performance metrics between all methods over 1,000 bootstrap samples, including FDR-adjusted *P* values, for the human cortex dataset (Trevino et al.).

Supplementary Dataset 5 Result of pairwise comparisons of performance metrics between all methods over 1,000 bootstrap samples, including FDR-adjusted *P* values, for the large human hematopoietic dataset (Satpathy et al.).

Supplementary Dataset 6 Result of pairwise comparisons of performance metrics between all methods over 1,000 bootstrap samples, including FDR-adjusted *P* values, for the human TME dataset (Satpathy et al.).

## Data Availability

For this study, we used only public datasets, available through the Gene Expression Omnibus: the small human hematopoietic dataset from GSE96769 and GSE74310; the mouse mammary epithelial dataset from GSE125523, in addition to processed files provided by the original study from https://github.com/jaychung10010/Mammary_snATAC-seq; the human cortex multiome dataset from GSE162170; the large human hematopoietic and TME datasets from GSE129785; and the large human fetal dataset from GSE149683. More details about downloading the raw and processed files for each dataset are described in the Supplementary Note.

## Code availability

CellSpace is freely available on Zenodo^25^ at 10.5281/zenodo.10521077 and on GitHub at https://github.com/zakieh-tayyebi/CellSpace. Instructions for installing and using CellSpace are provided in this GitHub repository, in addition to a tutorial, scripts and required data for training and interpreting a CellSpace model for the small human hematopoietic dataset. For this demo dataset, we have also provided preprocessing scripts and instructions to identify highly variable tiles and peaks using itLSI, which can be adapted to preprocess other scATAC-seq datasets. Details of preprocessing other datasets, running different methods on each dataset, all downstream analyses, computing performance metrics and bootstrapping the scores are provided in the Methods and Supplementary Note, and the scripts for reproducing these results are available upon request.
