# Supplementary material for: Scalable and unbiased sequence-informed embedding of single-cell ATAC-seq data with CellSpace
Source: Nat Methods. 2024 May 9;21(6):1014–22. doi: 10.1038/s41592-024-02274-x (PMC11166566; doi:10.1038/s41592-024-02274-x)
Supplement: Supplementary file 1 — Details about data preprocessing and running each model on different datasets. [file 41592_2024_2274_MOESM1_ESM.pdf]

nature methods

Article

https://doi.org/10.1038/s41592-024-02274-x

# Scalable and unbiased sequence-informed embedding of single-cell ATAC-seq data with CellSpace

In the format provided by the authors and unedited

## Supplementary Note

Dataset-specific preprocessing steps, CellSpace model hyperparameters, downstream analyses, and training details for competing embedding methods are described below.

### *Small-scale human hematopoiesis dataset*

Paired-end FASTQ files for 2,779 samples from GSE96769 and 192 LMPP/Mono samples from GSE74310[5] were downloaded using SRA toolkit v2.11. Adapters were detected and trimmed by TrimGalore v0.6.6 (FastQC and Cutadapt wrapper). Reads were aligned to hg19 by Bowtie2 v2.4.2 with '' --maxins 2000 --very-sensitive --no-unal --no-mixed". Barcodes were added to the aligned BAM files as 'CB' tags using a custom script. BAM files were filtered (MAPQ>30), coordinate-sorted, indexed, and merged by Samtools v1.11.

The final BAM file was inputted to ArchR, barcodes were filtered (TSS enrichment score > 4 and 5K < number of fragments < 200K), and doublets were detected and removed with default settings. 2,154 cells were retained for downstream analyses. Using ArchR's implementation of iterative LSI, the top 50K most variable tiles (genome-wide 500 bp bins) were identified after 5 iterations. This was restricted to the standard chromosomes chr1, …, chr22. The selected tiles and their corresponding cell-by-tile counts matrix were extracted for training a CellSpace model.

The result of scaling the dimensionality reduction from the last iteration of iterative LSI, named 'ArchR itLSI (var. tiles)', was used to build a NN graph and its SNN graph and to cluster the cells (method="Seurat") to identify ArchR clusters. The ArchR UMAP embedding was computed from the NN graph. The itLSI embedding was batch-corrected using Harmony[18] with 'Donor' annotations as batch labels.

A CellSpace model was trained with "--k 8 --sampleLen 150 --dim 30 --ngrams 3 --exmpPerPeak 20 --epoch 50" on the cell-by-tile count matrix. The resulting CellSpace embedding, named 'CellSpace (var. tiles)', was used for downstream analyses.

Pseudotime analysis was performed on the CellSpace embedding by standard functions from Palantir[12] v1.0.0. An HSC cell was manually selected as the starting point, but the potential trajectories and their terminal cells were identified by Palantir.

Moreover, we downloaded the peak atlas and cell-by-peak count matrix for the 2,154 cells, filtered as described previously, and retained 277,916 promoter-distal peaks from the standard chromosomes chr1, …, chr22 that were accessible in at least 5 cells.

The result of scaling the LSI reduced dimensions computed from the filtered count matrix was named 'LSI (peaks)'. The LSI embedding was batch-corrected using Harmony with 'Donor' annotations as batch labels.

Another CellSpace embedding, named 'CellSpace (peaks)', was trained with "--k 8 --sampleLen 150 --dim 30 --ngrams 3 --exmpPerPeak 20 --epoch 50" on the count matrix of the top 50K most variable peaks, which were identified by performing 5 iterations of iterative LSI. We did not correct for any possible batch effects during this process.

scBasset was run on the cell-by-peak matrix, with default settings, and the embedding was named 'scBasset (peaks)'. Peaks accessible in fewer than 5% of cells were removed per their default filtering. The embedding 'scBasset (batch-corrected)' was trained as described previously, with 'Donor' annotations as batch labels.

We performed different versions of SIMBA on the filtered count matrix. The peak-only embedding and the sequence-aware embedding were named ‘SIMBA (peaks)’ and ‘SIMBA (peaks+kmers+motifs)’, respectively. Furthermore, we computed a SIMBA embedding, named ‘SIMBA (batch-corrected)’, for which ‘Donor’ annotations were explicitly encoded as batch labels.

The PeakVI embedding, named ‘PeakVI (peaks)’, and the PeakVI embedding corrected for the ‘Donor’ batch effect, named ‘PeakVI (batch-corrected)’, were computed from the filtered count matrix.

We computed a chromVAR embedding using motifs, named ‘chromVAR (peaks+motifs)’, and one using DNA *k*-mers, named ‘chromVAR (peaks+*k*mers)’.

We compared the results, excluding the “unknown” cell type, as described in ‘*Evaluating scATAC-seq analysis results*’ (**Supplementary Fig. 2d,e** and **Supplementary Data Sets 2,3**). 'Cell type’ labels were used as ground truth to evaluate clustering results, and ‘Donor’ labels were used as batch labels (**Supplementary Fig. 2a-c**).

### *Mouse mammary epithelial dataset*

The promoter-distal peaks and the cell-by-peak raw count matrix were downloaded from https://github.com/jaychung10010/Mammary_snATAC-seq (GSE125523)[19]. The t-SNE embedding from the original study, computed from LSI reduced dimensions, was also downloaded (**Fig. 2f**).

The peaks from the standard chromosomes chr1, …, chr19, chrX that were in the upper 90% quantile in their total number of counts were retained for training a CellSpace model.

A CellSpace model was trained with “--k 8 --sampleLen 300 --dim 30 --ngrams 3 --exmpPerPeak 20 --epoch 20” on the 7,846 cell by 130,887 peak count matrix. We trained two additional CellSpace embeddings with “--ngrams 1” and --ngrams 5” and otherwise similar parameters.

### *Human cortex multiome dataset*

10X Multiome ATAC and RNA count matrices for human developing cerebral cortex sample (PCW21) were downloaded from GEO (GSE162170)[20]. The dataset consisted of 8,981 cells and 467,315 peaks with 14 identified cell types.

10X Multiome RNA data was processed with standard steps: genes expressed in fewer than 3 cells were discarded, counts were normalized to 10,000 reads per cell and then log transformed.

The LSI embedding was computed from the cell-by-peak count matrix.

A CellSpace embedding was trained with “--k 8 --sampleLen 150 --dim 30 --ngrams 3 --exmpPerPeak 10 --epoch 30”. Peaks not accessible in any cells were not included.

scBasset was trained as described previously. Peaks accessible in fewer than 5% of cells were removed per their default filtering, leaving 38,502 peaks. scBasset was trained for 45 epochs, at which point the validation loss and AUC had leveled and the correlation between the intercept and the library size was 0.99 (**Supplementary Fig. 3a**).

A peak-only embedding and a sequence-aware embedding were trained with SIMBA, as described previously.

The PeakVI embedding was trained on the count matrix with default settings.

Moreover, we computed a chromVAR embedding using JASPAR 2020 motifs.

'Cell type' labels were used as ground truth to evaluate clustering results, as described in '*Evaluating scATAC-seq analysis results*' (**Supplementary Fig. 3d,e** and **Supplementary Data Sets 2,4**).

To evaluate the transcription factor (TF) activity scores of each method for single-cell multiome data, we correlated the motif scores computed from the ATAC-seq readout with the gene expression of the corresponding TFs from the RNA-seq readout. For this evaluation, all methods used JASPAR 2020[29] as the motif database. Each TF may have multiple motifs in this database, so for each method, we selected the motif with the highest correlation to represent the TF.

Transcription factors important in cortical development, as identified in Figure 3F of Trevino *et al.*[20], were highlighted in red (PAX6, INSM1, SOX9, EMX2, LHX2, FOXG1, TCF4, TCF3, TFAP2C, FOS, POU3F3, NEUROD2, BHLHE22, JUND, MEF2C, POU2F2, NFIA, MEIS2, EOMES). Motifs were filtered for those present in peaks that were accessible in at least 5% of cells, due to the default peak filtering by scBasset.

### *Combination of multiple human cortex datasets*

The multiomic profiles of 8,981 human cortex cells, described in the previous section, as well as the scATAC-seq profiles of 31,304 human cortex cells, were downloaded from GEO (GSE162170)[20]. From the scATAC-seq dataset, we retained 12,675 cells from the time point present in the multiome dataset (post-conception week 21).

For each dataset, respectively, we identified the top 50K most variable peaks after 3 iterations of iterative LSI. The cell-by-peak count matrices of both datasets were used to train a co-embedding with CellSpace.

A CellSpace model was trained on both datasets simultaneously, with "--k 8 --sampleLen 150 --dim 50 --ngrams 3 --exmpPerPeak 20 --epoch 20". The resulting CellSpace embedding was used for downstream analyses, without any batch correction.

### *Large-scale hematopoietic and tumor microenvironment datasets*

Single-cell ATAC-seq profiles of human hematopoiesis and peripheral blood, consisting of 63,882 cells, and of human BCC TME, consisting of 37,818 cells, were downloaded from GEO (GSE129785)[21]. This includes 2,076 cells from the smaller human hematopoiesis dataset, previously described in '*Small-scale human hematopoiesis dataset'*.

The UMAP projection of the hematopoietic dataset from the original study, computed from LSI reduced dimensions with custom batch correction, was also downloaded (**Fig. 4a**).

For each dataset, respectively, we performed 5 iterations of iterative LSI to identify the top 50K most variable peaks. We did not correct for any possible batch effects during this process. The result of scaling the dimensionality reduction from the last iteration of iterative LSI was used as the itLSI embedding. The itLSI embedding was batch-corrected using Harmony with 'Source'/'Patient' annotations as batch labels.

A CellSpace model was trained with "--k 8 --sampleLen 150 --dim 70 --ngrams 3 --exmpPerPeak 100 --epoch 100" for each dataset, using the count matrix of top variable peaks.

scBasset was trained for 1000 epochs on the count matrix of top variable peaks, with default settings. The batch-corrected scBasset embedding was trained with ‘Source’/‘Patient’ annotations as batch labels.

A peak-only embedding, a sequence-aware embedding, and an embedding corrected for ‘Source’/’Patient’ batch effect were trained on the count matrix of top variable peaks with SIMBA. We did not restrict the training to peaks associated with top PCs.

The PeakVI embeddings with and without correction for ‘Source’/‘Patient’ batch effect were computed from the count matrix of top variable peaks as described previously.

We compared the results as described in ‘*Evaluating scATAC-seq analysis results*’ (**Supplementary Fig. 4d** and **Supplementary Data Sets 2,5,6**). 'Cell type’ labels (**Fig. 4b**, **Supplementary Fig. 4c**) were used as ground truth to evaluate clustering results. We used ‘Source’/‘Patient’ as batch labels (**Supplementary Fig. 4a,c**). The 4 tumor clusters were excluded from the evaluations for the TME dataset.

For 30,211 hematopoietic cells annotated as NK and T cells, we identified the top 50K most variable peaks and trained a CellSpace model with “--k 8 --sampleLen 150 --dim 30 --ngrams 3 --exmpPerPeak 50 --epoch 30”.

### *Large-scale human fetal dataset*

Single-cell profiles of chromatin accessibility with three-level combinatorial indexing (sci-ATAC-seq3) from 15 human fetal organs, consisting of 720,613 cells, were downloaded from GEO (GSE149683)[22].

To find variable peaks for this dataset, we sampled ~5% of cells from each organ (36,200 cells total), removed peaks that were accessible in <10 cells and peaks from chrY or chrM (leaving 1,048,007 peaks), and performed 5 iterations of iterative LSI to identify the top 100K most variable peaks of the down-sampled count matrix. We did not correct for any possible batch effects during this process.

A CellSpace model was trained for all ~720K cells and variable peaks, with “--k 8 --sampleLen 150 --dim 70 --ngrams 3 --exmpPerPeak 50 --epoch 300”.
