# Supplementary material for: Scalable and unbiased sequence-informed embedding of single-cell ATAC-seq data with CellSpace
Source: Nat Methods. 2024 May 9;21(6):1014–22. doi: 10.1038/s41592-024-02274-x (PMC11166566; doi:10.1038/s41592-024-02274-x)
Supplement: Supplementary file 2 — Reporting Summary [file 41592_2024_2274_MOESM2_ESM.pdf]

nature portfolio

Corresponding author(s): Tsutomu Suzuki, Matthew Waldor

Last updated by author(s): Mar 28, 2024

Reporting Summary

Nature Portfolio wishes to improve the reproducibility of the work that we publish. This form provides structure for consistency and transparency in reporting. For further information on Nature Portfolio policies, see our Editorial Policies and the Editorial Policy Checklist.

Statistics

For all statistical analyses, confirm that the following items are present in the figure legend, table legend, main text, or Methods section.

n/a	Confirmed	
☐	☒	The exact sample size (*n*) for each experimental group/condition, given as a discrete number and unit of measurement
☐	☒	A statement on whether measurements were taken from distinct samples or whether the same sample was measured repeatedly
☐	☒	The statistical test(s) used AND whether they are one- or two-sided<br>*Only common tests should be described solely by name; describe more complex techniques in the Methods section.*
☒	☐	A description of all covariates tested
☒	☐	A description of any assumptions or corrections, such as tests of normality and adjustment for multiple comparisons
☐	☒	A full description of the statistical parameters including central tendency (e.g. means) or other basic estimates (e.g. regression coefficient) AND variation (e.g. standard deviation) or associated estimates of uncertainty (e.g. confidence intervals)
☐	☒	For null hypothesis testing, the test statistic (e.g. *F*, *t*, *r*) with confidence intervals, effect sizes, degrees of freedom and *P* value noted<br>*Give P values as exact values whenever suitable.*
☒	☐	For Bayesian analysis, information on the choice of priors and Markov chain Monte Carlo settings
☒	☐	For hierarchical and complex designs, identification of the appropriate level for tests and full reporting of outcomes
☒	☐	Estimates of effect sizes (e.g. Cohen's *d*, Pearson's *r*), indicating how they were calculated

Our web collection on statistics for biologists contains articles on many of the points above.

Software and code

Policy information about availability of computer code

Data collection	Gel images and autoradiographs of membranes in binding assays were captured using FLA-7000 image analyzer (Fujifilm).<br>MS data were obtained by Xcalibur for LTQ Orbitrap XL (Thermo Fisher Scientific) and Q Exactive hybrid Quadrupole-Orbitrap mass spectrometer (Thermo Fisher Scientific).<br>Cryo-EM grids were prepared using Vitrobot Mark IV (Thermo Fisher Scientific).<br>Automated cryo-EM data acquisition was performed by EPU 2.9 software (Thermo Fisher Scientific) on a Krios G4 transmission electron microscope (FEI) equipped with a K3 direct electron detector (Gatan).<br>BioDrop resolution software version 3.3.6.0 (Biochrom) was used for UV data collection.<br>MassHunter Workstation Qualitative Analysis (Agilent)<br>SH800S Cell Sorter (Sony Biiotechnology)

Data analysis	Canvas X (version 20) and ChemDraw (20.1 and 22.2) were used to create figures.<br>UCSF Chimera (version 1.15) and UCSF ChimeraX (version 1.2) were used to analyze and prepare figures of cryo-EM maps and atomic models.<br>Microsoft Excel for Microsoft 365 MSO and R(3.4.3) was used for statistical analysis.<br>GraphPad Prism ver 7.04 and 9.3.1 were used to draw bar graphs of binding assay results.<br>Multi Gauge Version 3.0 was used to quantify the radioactivity in binding assays.<br>Qual Browser in Xcalibur 4.4 was used to analyze LC/MS data.<br>Phenix (1. 19. 2) and Coot (version 0.9.4) were used for model building.<br>RELION 3.1.2 and crYOLO (1.9.1) were used for cryo-EM image processing.<br>MassHunter Qualitative Analysis Navigator (Agilent, B.08.00)<br>SH800S software (Sony Biiotechnology)

For manuscripts utilizing custom algorithms or software that are central to the research but not yet described in published literature, software must be made available to editors and reviewers. We strongly encourage code deposition in a community repository (e.g. GitHub). See the Nature Portfolio guidelines for submitting code & software for further information.

Data

Policy information about availability of data

All manuscripts must include a data availability statement. This statement should provide the following information, where applicable:

- Accession codes, unique identifiers, or web links for publicly available datasets
- A description of any restrictions on data availability
- For clinical datasets or third party data, please ensure that the statement adheres to our policy

Publicly available datasets from Protein Data Bank (7K00, 4V8N, and 4V5R) were used for atomic model building and comparison.
Cryo-EM maps and atomic coordinates of the reported structures were deposited in Electron Microscopy Data Bank (EMDB) and Protein Data Bank, respectively, with the following accession codes; EMD-39577 and 8YUO (A-, P- site P.putida tRNAIle2 on AUAU mRNA); EMD-39578 and 8YUP (A-site P.putida tRNAIle2 on A4 mRNA); EMD-39579 and 8YUQ (A-site P.putida tRNAIle2 on dA4 mRNA); EMD-39580 and 8YUR (A-site P.putida tRNAIle2 on Am4 mRNA); and EMD-39581 and 8YUS (A-site P.putida tRNAIle2 on A(F)4 mRNA).

Research involving human participants, their data, or biological material

Policy information about studies with human participants or human data. See also policy information about sex, gender (identity/presentation), and sexual orientation and race, ethnicity and racism.

Reporting on sex and gender	This study does not involve human research.
Reporting on race, ethnicity, or other socially relevant groupings	This study does not involve human research.
Population characteristics	This study does not involve human research.
Recruitment	This study does not involve human research.
Ethics oversight	This study does not involve human research.

Note that full information on the approval of the study protocol must also be provided in the manuscript.

Field-specific reporting

Please select the one below that is the best fit for your research. If you are not sure, read the appropriate sections before making your selection.

☒ Life sciences ☐ Behavioural & social sciences ☐ Ecological, evolutionary & environmental sciences

For a reference copy of the document with all sections, see nature.com/documents/nr-reporting-summary-flat.pdf

Life sciences study design

All studies must disclose on these points even when the disclosure is negative.

Sample size	No sample size calculation was conducted. The sample size for cryo-EM analysis was determined on the basis of a overnight data collection to obtain sufficient number of particles and achieve high resolution of the complex.
Data exclusions	Particles in the poorly resolved classes after classification were excluded as is the standard practice in cryo-EM analysis. The particles showing low level of GFP or mCherry expression were excluded from the analysis in reporter assay.
Replication	A-site binding assay was performed in quintuplicate (n=3 or 5) to confirm the exact values of the binding ratio and all attempts were successful with consistent results as shown in the figures. Although cryo-EM analysis was not replicated, the atomic models were generated from thousands of micrographs. Fluorescent reporter assay was conducted with three replicates derived from different culture (n=3).

Randomization	Randomization is not relevant to this study since samples were not allocated into experimental groups in this study.
Blinding	Blinding is not relevant to this study since the results cannot be affected by whether sample identities were disclosed.

Reporting for specific materials, systems and methods

We require information from authors about some types of materials, experimental systems and methods used in many studies. Here, indicate whether each material, system or method listed is relevant to your study. If you are not sure if a list item applies to your research, read the appropriate section before selecting a response.

Materials & experimental systems

n/a	Involved in the study	
☒	☐	Antibodies
☐	☒	Eukaryotic cell lines
☒	☐	Palaeontology and archaeology
☒	☐	Animals and other organisms
☒	☐	Clinical data
☒	☐	Dual use research of concern
☐	☒	Plants

Methods

n/a	Involved in the study	
☒	☐	ChIP-seq
☒	☐	Flow cytometry
☒	☐	MRI-based neuroimaging

Eukaryotic cell lines

Policy information about cell lines and Sex and Gender in Research

Cell line source(s)	Nicotiana tabacum BY-2 cell line was obtained from Riken BioResource Research Center.
Authentication	BY-2 cells are the suspension culture cells that are most widely used in plant science as a model plant system. BY-2 cells obtained from RIKEN BRC was directly used.
Mycoplasma contamination	Not applicable.
Commonly misidentified lines (See ICLAC register)	None.

Dual use research of concern

Policy information about dual use research of concern

Hazards

Could the accidental, deliberate or reckless misuse of agents or technologies generated in the work, or the application of information presented in the manuscript, pose a threat to:

No	Yes	
☒	☐	Public health
☒	☐	National security
☒	☐	Crops and/or livestock
☒	☐	Ecosystems
☒	☐	Any other significant area

Experiments of concern

Does the work involve any of these experiments of concern:

No	Yes	
☒	☐	Demonstrate how to render a vaccine ineffective
☒	☐	Confer resistance to therapeutically useful antibiotics or antiviral agents
☒	☐	Enhance the virulence of a pathogen or render a nonpathogen virulent
☒	☐	Increase transmissibility of a pathogen
☒	☐	Alter the host range of a pathogen
☒	☐	Enable evasion of diagnostic/detection modalities
☒	☐	Enable the weaponization of a biological agent or toxin
☒	☐	Any other potentially harmful combination of experiments and agents

Plants

Seed stocks	Arabidopsis thaliana Col-0 was cultivated by Inplanta Innovations Inc. Their seed stock was used.
Novel plant genotypes	No novel plant genotypes were produced.
Authentication	n/a
